# It Is All about (U)biquitin: Role of Altered Ubiquitin-Proteasome System and UCHL1 in Alzheimer Disease

**DOI:** 10.1155/2016/2756068

**Published:** 2016-01-05

**Authors:** Antonella Tramutola, Fabio Di Domenico, Eugenio Barone, Marzia Perluigi, D. Allan Butterfield

**Affiliations:** ^1^Department of Biochemical Sciences, Sapienza University of Rome, Italy; ^2^Facultad de Salud, Universidad Autónoma de Chile, Instituto de Ciencias Biomédicas, Providencia, Santiago, Chile; ^3^Department of Chemistry, Sanders-Brown Center of Aging, University of Kentucky, Lexington, KY 40506, USA

## Abstract

Free radical-mediated damage to macromolecules and the resulting oxidative modification of different cellular components are a common feature of aging, and this process becomes much more pronounced in age-associated pathologies, including Alzheimer disease (AD). In particular, proteins are particularly sensitive to oxidative stress-induced damage and these irreversible modifications lead to the alteration of protein structure and function. In order to maintain cell homeostasis, these oxidized/damaged proteins have to be removed in order to prevent their toxic accumulation. It is generally accepted that the age-related accumulation of “aberrant” proteins results from both the increased occurrence of damage and the decreased efficiency of degradative systems. One of the most important cellular proteolytic systems responsible for the removal of oxidized proteins in the cytosol and in the nucleus is the proteasomal system. Several studies have demonstrated the impairment of the proteasome in AD thus suggesting a direct link between accumulation of oxidized/misfolded proteins and reduction of this clearance system. In this review we discuss the impairment of the proteasome system as a consequence of oxidative stress and how this contributes to AD neuropathology. Further, we focus the attention on the oxidative modifications of a key component of the ubiquitin-proteasome pathway, UCHL1, which lead to the impairment of its activity.

## 1. Introduction

The physiological aging process and age-related diseases share many common features among which are accumulations of oxidative damage, impaired mitochondrial activity, and reduced efficiency of clearance systems among others. In particular, the reduced activity of the “quality control system” (PQC), including the ubiquitin-proteasome system, autophagy, and other intracellular proteolytic enzymes, leads to the accumulation of oxidized/unfolded proteins that may contribute to neuronal loss. Deposits of aggregated, misfolded, and oxidized proteins accumulate normally over the lifespan in cells and tissues and enormously increase in neurodegenerative diseases [1]. Insoluble aggregates can be formed as a result of covalent cross-links among peptide chains, as in the case of amyloid-*β*-peptide (A*β*) in Alzheimer disease (AD), *α*-synuclein in Parkinson disease (PD), huntingtin in Huntington disease (HD), and SOD1 in amyotrophic lateral sclerosis (ALS).

Oxidative modification of a protein represents one of the major causes of its increased susceptibility to aggregate. Indeed, proteins are sensitive to oxidative stress-induced chemical modifications, undergoing several structural changes that are not always correctly recognized by the proteasome, thus generating impaired protein function. The balance between functional proteins, present in young/healthy cells, and damaged or altered proteins, present at higher concentration in aged/diseased cells, depends mainly on their modification and turnover [2]. It is generally accepted that the age-related accumulation of “aberrant” proteins results from both the increased occurrence of damage and the decreased efficiency of degradative systems. Clearance of oxidatively modified proteins most often occurs through the proteasome system. The proteasome is the principal pathway to remove senescent and damaged proteins, and intact proteasome function is essential to preserve cellular homeostasis during oxidative stress conditions [3]. The age-related impairment of proteasome function in different cell types and organs has been widely demonstrated [4]. Such a decline of proteasome activity would therefore be expected to promote the accumulation of oxidized proteins with age. The reduced activity of the proteasome and other intracellular proteolytic machineries also has significant implications in the development of neurodegenerative diseases [4, 5].

What is the picture if oxidative stress targets members of the PQC? A number of studies suggest that oxidative stress can target the proteasome and impair its ability to correctly degrade oxidized proteins (reviewed in [6]). Further, ubiquitin-immunopositive inclusion bodies are commonly detected in the brain of patients suffering neurodegenerative diseases possibly as a result of impaired proteasome activity [4]. If from one side low levels of ROS are able to activate the expression of inducible proteasome subunits, at increasing ROS concentrations the proteasome subunits are susceptible to undergoing oxidative modifications [7], which ultimately result in impaired proteasome function. In addition, nondegradable protein aggregates and cross-linked proteins are able to bind to the proteasome, which makes the degradation of other misfolded and damaged proteins less efficient.

Intriguingly, recent studies proposed the impairment of proteasome activates autophagy, which might be a compensatory mechanism allowing eliminating ubiquitin-proteasome system (UPS) substrates [8, 9]. Indeed, treatment of both cells and mice with rapamycin, to induce autophagy, was able to protect against cell death caused by proteasome inhibition [10] and to protect against genetic loss of proteasome activity in* Drosophila* [8]. However, the exact mechanisms of the cross-talk between proteasome and autophagy are still not well understood. Among proposed mechanisms the activation of endoplasmic reticulum (ER) stress, due to the accumulation of misfolded proteins that leads to the induction of the unfolded protein response (UPR), is an interesting candidate. These different mechanisms may not be mutually exclusive and may also be of different importance in different cell types or at different time-points after the proteasome is inhibited [11].

In this review, we focus attention on the impairment of the proteasome system as a consequence of oxidative stress and how this impairment contributes to neurodegeneration. We suggest that reduced protein turnover may be caused by the selective oxidative damage of members of the proteasomal system that once targeted by oxidative stress are not able to fulfil their protective roles and contribute to the dysregulation of intracellular protein homeostasis. The complex interactions of these events in cellular protein and redox homeostasis in the brain are essential to design novel therapeutic intervention that may possibly retard the development of AD and other neurodegenerative diseases. AD is a disorder that leads to cognitive, behavioral, and memory deficits. The hallmarks of AD are the accumulation of A*β* into senile plaques and hyperphosphorylated tau into neurofibrillary tangles which consequent neuronal loss in select brain areas involved in learning and memory. A*β* is cleaved from amyloid-*β* protein precursor (APP) and comprises a set of 39–43 residue polypeptides that exert a range of neurotoxic effects that are considered to be important to the evolution of the pathology.

**Figure 1:**
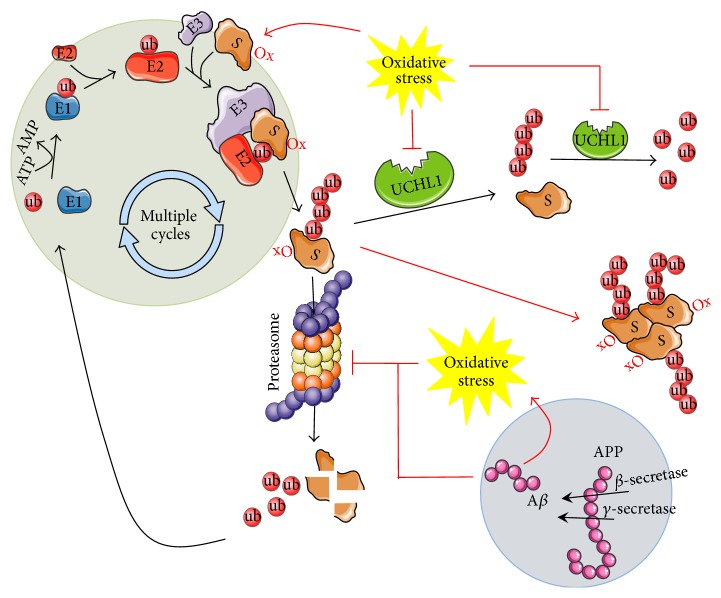
Oxidative stress impairs the functionality of the UPS in AD. Under physiological conditions the ubiquitin-proteasome system (UPS) mediates the clearance of misfolded proteins in order to prevent their toxic accumulation. Indeed, a target protein undergoes multiple cycles during which it is conjugated with one or more ubiquitin groups leading to mono- or polyubiquitinylated substrates (S). In particular, in the first step of this cycle, ubiquitin activating enzyme (E1) forms a thioester bond with ubiquitin and this reaction requires ATP as driving force. Subsequently, the ubiquitin group is transferred to ubiquitin-conjugating enzyme (E2), which works as a scaffold protein favoring the interaction between ubiquitin ligase (E3) and the target substrate, aimed at allowing the ligase to transfer the ubiquitin group from E2 to the substrate. After a number of cycles promoting the polyubiquitinylation of the substrate, this latter is driven to the proteasome for its degradation. Polyubiquitinylated substrates also can be targets of the activity of the ubiquitin carboxyterminal hydrolase L1 (UCLH1), which is highly expressed in neurons and hydrolyses small adducts of ubiquitin to generate the ubiquitin monomer. During the progression of Alzheimer disease (AD), increased amyloid-beta (A*β*) production and accumulation favor the augmentation of oxidative stress levels, which leads to protein oxidative modifications (Ox). Because oxidized proteins are neurotoxic, they would be eliminated through the UPS, but this does not seem to be the case in AD. Indeed, both A*β* and oxidative stress would promote the impairment of the UPS and the consequent accumulation of polyubiquitinylated proteins, which are visible as aggregates in AD brain. Arrows, promotion; lines, inhibition. Black, physiological conditions; red, pathological conditions.

*Ubiquitin-Proteasome System*. In the case of protein misfolding and aggregation in cells, the PQC system uses three main parallel strategies to maintain protein homeostasis. The misfolded protein may be refolded to recover the protein's normal conformation. Different molecular chaperones, such as heat shock proteins (HSPs), play essential roles in protein refolding. Alternatively, if the protein cannot be refolded, it is targeted to the UPS or autophagy programs for degradation (Figure 1) [12].

The UPS is located in the cytosol and the nucleus, and it is responsible for the degradation of more than 70–80% of intracellular proteins. The UPS degrades misfolded/damaged proteins and removes proteins involved in many cellular processes, such as signal transduction, cell cycle regulation, and cell death, and, ultimately, regulates gene transcription [13, 14].

Most of the proteins targeted for proteasome degradation are covalently modified by ubiquitin, often in a polyubiquitin chain. Ubiquitin is an 8.5 kDa protein composed of 76 amino acids. The ubiquitin protein is transcribed from* UBB*,* ubiquitin C (UBC)*,* RPS27A*,* and UBCEP2* genes. However, only the first two genes encode for a polyubiquitin precursor that is involved in the UPS signaling cascade. The other genes encode ubiquitin that fuses to ribosomal proteins [15]. Ubiquitin can form polyubiquitin chains at seven lysine residues on the target protein: Lys-6, Lys-11, Lys-27, Lys-29, Lys-33, Lys-48, and Lys-63. These chains are formed by the successive attachment of monomers by an isopeptide bond, most frequently formed between the side chain of Lys-48 in one ubiquitin and the carboxyl group of the C-terminal Gly-76 of a neighbouring ubiquitin. Attachment of Lys-48 polyubiquitin chains to lysine residues on a protein results in at least a 10-fold increase in its degradation rate [16]. Polyubiquitin chains with linkages involving lysine residues on ubiquitin other than Lys-48 were found to play distinct roles. Ubiquitin is conjugated through the formation of an isopeptide bond between the *ε*-amino group of a lysine residue of the substrate and the C-terminal carboxylate [17]. First, ubiquitin needs to be activated by an E1 enzyme in an ATP-dependent reaction, which results in a high-energy thioester bond between the E1's active site cysteine and the carboxyl group of the ubiquitin protein. Then, E2 (ubiquitin-conjugating enzyme) receives ubiquitin from E1 and forms a similar thioester intermediate with ubiquitin. E3 (ubiquitin ligase) binds both E2 and the substrate and transfers the ubiquitin to the substrate [18]. The E3 ligase is a pivotal enzyme in the UPS cascade and is crucial for substrate specificity. E3 plays a critical role in selecting the substrates, and the regulation of either its catalytic activity or substrate interaction properties is important for the UPS signaling pathway. E3 ligase enzymes can be grouped into two classes: those that are homologous to the E6-AP carboxyl terminus (HECT) and the gene RING ligases. The two classes differ not only in their structure but also in the way they catalyze the last step of ubiquitinylation. The HECT ligases accept the activated ubiquitin from an E2 enzyme on a cysteine residue in the active domain and then transfer it to the substrate, whereas the RING ligases act as scaffold proteins by bringing together an E2 conjugating enzyme and the substrate [19]. The F-box and leucine rich repeat protein 2 (FBL2) is another component of the SCF (Skp1–Cullin1–F-box protein) E3 ubiquitin ligase complex that has been found to be decreased in the brains of AD patients [20]. Interestingly, the Watanabe group demonstrated that FBL2 impacts APP metabolism by interacting with APP to modulate APP ubiquitinylation. In detail, FBL2-mediated ubiquitinylation of APP inhibits its endocytosis [21].

In some circumstances, a fourth ubiquitinylation enzyme, known as the ubiquitin chain elongation factor E4, is necessary, together with the E1, E2, and E3 enzymes, to extend a polyubiquitin chain [22]. This cascade results in ubiquitinylation of the target protein. A polyubiquitin chain of at least four ubiquitin moieties is necessary for efficient translocation of the substrate to the proteasome [23]. Monoubiquitinylation is not associated with protein degradation, but with endocytosis, protein sorting, DNA damage response, and epigenetics [24–26].

Ubiquitinylation is a reversible posttranslational modification, and a family of proteases, the deubiquitinylating enzymes (DUBs), can remove ubiquitin from substrates, thereby regulating the ubiquitinylation process and recycling ubiquitin. The recycling of ubiquitin is critical for the brain, which has a fixed amount of ubiquitin. DUBs are highly specific and have been grouped into five subfamilies: ubiquitin carboxyl-terminal hydrolases (UCH), ubiquitin-specific proteases (USP), ovarian tumor- (OTU-) like proteases, JAB1/MPN/Mov34 (JAMM/MPN) metalloproteases, and the Machado-Jakob disease proteases [27]. The state of substrate ubiquitinylation depends on the balance between ubiquitinylating and deubiquitinylating enzymes acting on a protein. Thus, cells developed a highly dynamic strategy based on a switch-on/switch-off type of mechanism that responds promptly to cellular requirements for proteolysis by the UPS. Ubiquitinylated substrates with a Lys-48-linked ubiquitin chain of sufficient length are targeted to the 26S proteasome for degradation.

The 26S proteasome is composed of a 20S catalytic core and two 19S regulatory caps on both ends of the 20S core. The 20S proteasome contains four stacked rings that form a barrel-shaped moiety with a central cavity [28]. These stacked rings include two noncatalytic outer rings called *α*-rings and two catalytic inner rings called *β*-rings. Three proteolytic activities are confined to the *β*-rings including chymotrypsin-like, caspase-like, and trypsin-like protease activities [19]. The 19S caps contain at least 18 subunits, with a base composed of six ATPases that exert a chaperone-like activity and a lid composed of eight subunits that recognize the polyubiquitin signals. The 19S proteasome binds and unfolds ubiquitinylated proteins and opens the entry gate of the 20S proteasome to allow protein in the central cavity [19].

Oxidation of a protein induces several reversible or irreversible alterations, including amino acid modification, fragmentation, or aggregation and causes increased susceptibility of the modified proteins towards proteolysis [29, 30]. It has been suggested that the oxidation of proteins causes the exposure of hydrophobic moieties to the surface via partial unfolding which are targeted by proteasome [31–33]. While the 26S proteasome degrades polyubiquitinylated proteins, the 20S proteasome by itself seems to be sufficient to degrade nonubiquitinylated oxidatively modified proteins in an ATP-independent manner; however, the exact mechanism is still unclear [13, 18, 19].

*Deubiquitinylating Enzymes: UCHL1*. DUBs function in the processing of ubiquitin precursors and ubiquitin adducts [34]. DUBs belong to a protease superfamily, and about 100 members are expressed in humans [35, 36]. The UCH class of DUBs consists of four proteins (Bap1, UCHL1, UCHL3, and UCHL5), which all have a conserved catalytic domain (UCH-domain) consisting of about 230 amino acids [35, 36]. Ubiquitin carboxyterminal hydrolase L1 (UCHL1) is a 223-amino-acid protein encoded by 9 exons [37]. The catalytic area of UCHL1 has a loop positioned over the active site, which limits the size of ubiquitin adducts that can be processed by it to small peptides [38]. UCHL1 is proposed to function largely by maintaining a stable pool of monoubiquitin for use in ubiquitinylation reactions, as showed in Figure 1 [27, 35]. Newly translated ubiquitin contains amino acids following the terminal glycine residue that is used for isopeptide bond formation. UCHL1 can cleave off these additional amino acids in order to expose the final glycine of ubiquitin for conjugation. UCHL1 can also help maintain the monoubiquitin pool by reversing accidental modifications that can form during ubiquitin activation [39]. Due to its specificity, UCHL1 does not remove ubiquitin from all proteins. It is this selectivity that offers unique advantages for drug discovery efforts. UCHL1 is also involved in the cotranslational processing of proubiquitin and ribosomal proteins translated as ubiquitin fusions [37]. In addition, UCHL1 can form dimers, whose form seems to act as another enzymatic activity in UCHL1, the ubiquitin ligase activity [40]. In the dimeric form, UCHL1 ligase activity produces Lys-63-linked ubiquitin chains to its substrates. In contrast to the well-recognized ubiquitinylation pathway with E1, E2, and E3 ligases, UCHL1 does not require ATP as a notable characteristic of this ligase. Interestingly, when they are polyubiquitinylated via Lys-63 of ubiquitin, the substrates escape from UPS-dependent protein degradation leading to their stabilization. The dual function of both addition and removal of monoubiquitin sets UCHL1 apart from other DUBs and makes it a special target for proper UPS function [41].

UCHL1 is among the most abundant proteins in the brain reaching 1-2% of total brain lysate and regulating the timing and the pattern of ubiquitinylation of brain proteins [37]. It is also one of the main enzymes that play a role in maintaining free ubiquitin levels in neurons [20, 21]. UCHL1 is also present in the peripheral nervous system, such as the dorsal root ganglion and trigeminal ganglion neurons [36]. UCHL1 is involved in synaptic activities and a reduction in UCHL1 function has been linked to neurodegenerative diseases [36, 42]. UCHL1 is also studied due to its association with various malignancies, including colorectal, breast, prostate, and lung cancers [43]. The* UCHL1* gene is known as* PARK5*, and its mutations are associated with Parkinson disease [42]; indeed the I93M mutation shows severely diminished hydrolase activity and lower E3 activity, while the S18Y mutant has greater hydrolase activity but lower E3 activity than WT [37, 44]. Further, deletion of one of the active site residues of the* UCHL1* gene is associated with gracile axonal dystrophy and leads to elevated oxidative damage in the brain [45].

UCHL1 is susceptible to oxidative damage and when this occurs it has aberrant functions analogous to mutated UCHL1 [46, 47]. Moreover, aberrant UCHL1 is able to interact with Lamp2a, Hsc70, and Hsp90 thus inhibiting chaperone mediated autophagy- (CMA-) dependent degradation and causing the accumulation of CMA substrates (e.g., *α*-synuclein) [48, 49].

## 2. Impairment of Ubiquitin-Proteasome System in Alzheimer Disease

The first observations about increased ubiquitin accumulation in specific structures characterizing human AD brain were showed about 30 years earlier. Indeed, ubiquitin was found to be covalently associated with the insoluble material of NFT and SP [50–52], thus suggesting that something in the degradative systems did not work properly. However, the presence of ubiquitin in abnormal aggregates was not associated with the subsequent proteolytic step [51]. Specifically, A*β* inhibited the chymotrypsin-like activity but had no effect on the proteolytic activity of the protease chymotrypsin, suggesting that A*β* did not interact with the active site of the proteasome subunit [53]. Reduced chymotrypsin-like and peptidyl-glutamyl peptide-hydrolysing activity was found in AD brain [54].

The role of A*β* peptides in those processes was further investigated by Oh et al., who demonstrated that increased A*β* levels paralleled decreased chymotrypsin-like activity of the 26S proteasome in cortex and hippocampus of Tg2576 mice, a well characterized AD animal model that ubiquitously expressed Swedish mutant amyloid precursor protein (APPswe) [55]. These results were also confirmed in B103 cells, a rat neuroblastoma cell line, in which A*β* treatment led to the inhibition of the proteasome activity [55], even though the mechanism involved in the inhibition of proteasome induced by A*β* is still unclear.

Because the effects mediated by A*β* can be dependent on its aggregation state, Cecarini and colleagues analyzed the impact of nonfibrillar, oligomeric, and fibrillar forms of A*β* on the proteasome activities in both the isolated 20S proteasome and SH-SY5Y cells [56]. They found a significant reduction only in the chymotrypsin-like activity in isolated 20S proteasome preparations treated with A*β*, independent of the aggregation state of this peptide [56]. Rather, these investigators showed a general decrease of the proteasome functionality especially upon treatment with the oligomeric and fibrillar forms in SH-SY5Y cells [56]. In fact, comparing these assays with that obtained using purified proteasomes, the tested activities were all significantly reduced. The marked decrease in proteasome functionality was also confirmed by the enhancement in the levels of ubiquitin protein conjugates. These results agree with the proposed toxic role of A*β*, possibly independent of its aggregation state.

While, on one hand, A*β* could be directly responsible for the proteasome impairment as cited above, on the other hand, the impairment of other members belonging to the UPS could also favor A*β* accumulation. In light of these findings A*β* is a part of a vicious cycle whereby its accumulation promotes the proteasome impairment responsible for further accumulation of A*β*-proteins.

Interestingly, Rosen et al. reported that the overexpression of the ubiquitin E3 ligase Parkin, which was found reduced in human AD brain, greatly decreased the levels of intracellular A*β*-42 in neurons [57]. This effect was abrogated by proteasome inhibition [57]. In addition, these researchers reported that intracellular A*β*-42 accumulation decreased cell viability and proteasome activity, while Parkin reversed both effects [57]. The importance of Parkin has been further highlighted in a subsequent study, where the overexpression of Parkin in APP/PS1 transgenic mice restored activity-dependent synaptic plasticity and rescued behavioral abnormalities [58].

Similarly, the ubiquitin ligase HRD1, which normally promotes APP ubiquitinylation and degradation resulting in decreased generation of A*β*, was found impaired in AD brain [59]. Indeed, suppression of HRD1 induced APP accumulation and increased production of A*β in vitro*, resulting in apoptosis [59]. In addition, Zhang et al. found that inhibition UCHL1 significantly increased *β*-secretase 1 (BACE1) protein level* in vitro *[60]. BACE1 half-life was reduced in cells overexpressing UCHL1 and decreased APP C-terminal fragment C99 and A*β* levels were observed [60].

Taken together, the impairment of members of the UPS different from the 26S proteasome itself, such as Parkin, HRD1, and UCHL1, may affect APP processing and A*β* production.

### 2.1. Oxidative Damage to UPS

Among the factors contributing to the impairment of UPS in AD, augmentation of the oxidative/nitrosative stress levels was proposed as conceivable causative effect [61]. Indeed, levels of oxidized proteins in AD are associated with loss of the activity of the 20S proteasome, which, as noted above, represents a major enzyme for the degradation of oxidized proteins [54, 62–64]. Interestingly, studies from the Davies and Grune groups showed that moderately oxidized proteins are preferentially recognized and degraded by the proteasome; however, severely oxidized proteins cannot be easily degraded and, instead, inhibit the proteasome [3, 65]. Further, studies have shown that prolonged oxidized proteins are more resistant to degradation by 20S proteasomes [66, 67]. Therefore, overloading the UPS by undegradable substrates, mutations, or oxidative damage may lead to the accumulation of abnormal proteins and to the selective degeneration of neurons.

In that context both the Butterfield group [46, 68] and others [69] demonstrated that UCLH1 is oxidatively modified in AD, establishing a link between the effect of oxidative stress on protein and the proteasomal dysfunction. Similarly, Saito et al. showed that HRD1 protein was insolubilized by oxidative stress but not by other AD-related molecules and stressors, such as amyloid-*β*, tau, and ER stress. Furthermore, these authors raised the possibility that modifications of HRD1 by 4-hydroxy-2-nonenal, decreased HRD1 protein solubility leading to the accumulation of HRD1 into the aggresome [70]. In addition, the identification of oxidative stress-induced modification of the heat shock cognate 71 seems to underlie the essential link between the folding and degradation machineries that once impaired by oxidative damage become critical for cell viability [71].

Nitric oxide- (NO-) induced S-nitrosylation of the protein disulphide isomerase (PDI) was proposed to have a role relating protein misfolding to neurodegeneration [72]. Indeed, S-nitrosylation inhibited PDI enzymatic activity and led to the accumulation of polyubiquitinylated proteins [72, 73]. S-nitrosylation also abrogated PDI-mediated attenuation of neuronal cell death triggered by ER stress, misfolded proteins, or proteasome inhibition [72]. Thus, PDI prevents neurotoxicity associated with ER stress and protein misfolding, but NO blocks this protective effect in neurodegenerative disorders through the S-nitrosylation of PDI [72].

Cecarini et al. also demonstrated that despite lack of differences in the amount of proteasome complex isolated from control, MCI, and AD brains, a large impairment in proteasome-mediated degradation of an oxidized protein was observed in MCI and AD subjects [74]. The impairment was associated with the elevation of proteasome oxidative modifications such as protein carbonyls, 4-hydroxynonenal-conjugation, and neuroprostane-conjugation [74]. Intriguingly, the incubation of proteasome complexes with a reducing agent fully restored proteasome-mediated protein degradation in both MCI and AD samples, thus supporting a role for oxidative stress in promoting proteasome inactivation [74].

### 2.2. Mutant Ubiquitin UBB^+1^

Together with A*β* and oxidative/nitrosative stress, a mutant form of ubiquitin, deriving from a molecular misreading of the* ubiquitin* gene and termed UBB^+1^, was found to be selectively expressed in the brains of AD patients [75] and was reported to impair the proteasome activity* in vitro *[76].

Indeed, Lam and colleagues showed for the first time that (i) UBB^+1^ is polyubiquitinylated (UBB^+1^-polyubiquitin); (ii) UBB^+1^-polyubiquitin was strongly resistant to disassembly and accumulates in cells; and (iii) UBB^+1^-polyubiquitin inhibited proteasomal activity, thus providing a likely mechanism of toxicity [76]. In accordance with these observations, overexpression of UBB^+1^ in neuroblastoma cells significantly induced nuclear fragmentation and cell death [77].

The complex nature of UBB^+1^ interactions was illustrated by the finding that on one hand the induction of UBB^+1^ expression in SH-SY5Y cells caused proteasome inhibition, while on the other hand UBB^+1^ also induced the expression of heat shock proteins, which conferred a subsequent resistance to tertbutyl hydroperoxide-mediated oxidative stress. Indeed, these authors concluded that although UBB^+1^-expressing cells have a compromised ubiquitin-proteasome system, these cells are protected against oxidative stress conditions. However, which one of these two effects is prevalent does not emerge from the study and requires further investigations [78].

From the point of view of the mechanisms underlying UBB^+1^-induced neurotoxicity, an ubiquitin-conjugating enzyme, E2-25K/Hip-2, which was found to be upregulated in the neurons exposed to A*β*42 and in the brain of AD patients, was proposed to have a role [76, 79]. E2-25K/Hip-2 seems to function both as an E2 ubiquitin-conjugating enzyme like other E2 proteins and as an unusual ubiquitin ligase to produce diubiquitin and unanchored polyubiquitin chains without any E3 ligase [80]. E2-25K/Hip-2 was shown to reduce proteasome activity [79]. Rather, E2-25K/Hip-2 was found to play a major role in A*β* neurotoxicity by promoting the polyubiquitinylation of UBB^+1^, which leads to proteasome inactivation [79].

However, a protective role for UBB^+1^ was also reported. By using a triple transgenic mouse (APP/PS1/UBB^+1^) obtained by crossing UBB^+1^ and APP/PS1 transgenic mice, van Tijn and colleagues showed a transient and significant decrease in A*β* deposition and soluble A*β* 1–42 levels in APP/PS1/UBB^+1^ transgenic mice compared to APP/PS1 mice at 6 months of age [81].

### 2.3. Dysfunction of UPS and Tau Aggregation

While A*β* was mainly found to both directly and indirectly trigger the inhibition of proteasome activity, probably the most investigated target in terms of proteins aggregation following proteasome inhibition in AD is tau. Studies in AD brain demonstrated that phosphorylated tau accumulated on both sides of the synapse, thus showing synaptic enrichment of this protein when compared with the cytoplasm [82]. The accumulation of p-tau at the synapse mirrors the accumulation of ubiquitinylated proteins in the same fraction, as well as the accumulation of proteasomes and related chaperones, consistent with the notion that tau aggregates are associated with impaired proteolysis mediated by the UPS [82].

Zhang et al. in 2005 reported for the first time that tau, both phosphorylated and nonphosphorylated, is degraded by the 26S proteasome in an ubiquitin- and ATP-dependent manner, suggesting that defect in the UPS would promote tau accumulation [83].

In agreement with the above, Cripps et al. reported that soluble paired helical filaments (PHF) of tau protein are ubiquitinylated at their microtubule-binding domain (at residues Lys-254, Lys-311, and Lys-353), suggesting that ubiquitinylation of PHF-tau may be an earlier pathological event and that ubiquitinylation could play a regulatory role in modulating the integrity of microtubules during the course of AD [84]. Through the use of tandem mass spectrometry, the same group highlighted that PHF-tau is modified by three polyubiquitin linkages at Lys-6, Lys-11, and Lys-48 [84]. Among these, Lys-48-linked polyubiquitinylation is the primary form of polyubiquitinylation with a minor portion of ubiquitin linked at Lys-6 and Lys-11 [84]. Because modification by Lys-48-linked polyubiquitin chains is known to serve as the essential means of targeting proteins for degradation by the ubiquitin-proteasome system, a failure of the UPS could play a role in tau accumulation in AD [84].

The role of PHF-tau was further highlighted by Gillardon et al., who proposed that the reduced peptidase activity observed in AD brain extracts is not an intrinsic property of the 20S proteasome but may be resulting from the presence of endogenous inhibitory proteins or substrates, for example, PHF-tau [85]. Indeed, these investigators found that proteasome activity was increased upon purification from AD brain [85], while the presence of cytosolic proteins, which had been removed during the purification process, led to proteasome inhibition [85].

Quite recently, HRD1 ubiquitin ligase, previously reported to favor APP degradation [59], was also identified as a negative regulator of tau phosphorylation in AD [86]. In fact, Shen et al. reported that HRD1 interacts with tau and promotes the degradation of both dephosphorylated and phosphorylated tau through the 26S proteasome [86].

An intriguing aspect about proteasome-mediated degradation of tau protein was the discovery that hyperphosphorylation of tau diminishes its recognition by the proteasome [87], thus questioning which form of proteasome is responsible for tau degradation: the classical ATP/ubiquitin-dependent 26S proteasome pathway and/or a 20S proteasome pathway not requiring ubiquitin or ATP? Indeed, the Poppek group reported that the ATP/ubiquitin-independent 20S proteasome could degrade tau* in vitro* [87], while evidence also exists that, under certain conditions, the tau protein is polyubiquitinylated and directed to the 26S proteasome [83, 84, 88, 89]. Starting from such apparently conflicting reports, Grune and colleagues demonstrated* in vitro* that the normal turnover of the tau protein is catalyzed by the proteasome in an ATP/ubiquitin-independent manner and that the 20S proteasome is more important for normal tau turnover than is the 26S proteasome [90]. This interpretation seems reasonable, since the tau protein is largely unfolded and, therefore, should not require ATP for unfolding prior to degradation [90]. Conversely, under other conditions, including certain stress situations, the ATP/ubiquitin pathway and the 26S proteasome may be more important [90].

### 2.4. Role of ATP and Aggregated Proteins

Because ATP represents the essential driving force for UPS activity links between mitochondrial impairment and proteasome activity also have been evaluated. Huang et al. in 2013 demonstrated that cortical neurons treated with inhibitors of different elements of the electron transport chain showed a reduction in ubiquitinylated proteins and E1 activity as well as a calpain-mediated disassembly of the 26S proteasome [91]. Calpain activation promoted the cleavage of the microtubule-associated protein tau, leading to its accumulation [91]. Furthermore, all these changes paralleled increased 20S proteasome levels and activity [91]. The concomitant rise in the 20S proteasomes, which seem to degrade proteins in an unregulated and energy-independent manner, in the short-term may carry out the turnover of randomly unfolded oxidized proteins [91]. However, if chronic, this process could lead to neurodegeneration, as regulated protein degradation by the ubiquitin/proteasome pathway is essential for neuronal survival [91].

Although polyubiquitin aggregates are evident in AD brain, the identification of the proteins present in that structure is still elusive and studies on this are ongoing in our laboratories. Indeed, only a few examples emerge from the literature aimed at strengthening the role of the impaired UPS response in the progression of AD pathology.

*β*-Catenin, a member of the Wnt-signaling pathway, is a multifunctional protein that participates in cadherin-mediated cell adhesion and in transcriptional activation of Wnt target genes involved in development [92]. In the absence of a Wnt ligand, *β*-catenin is phosphorylated and is targeted for multiubiquitinylation by *β*-Trcp E3 ligase followed by rapid degradation by the 26S proteasome machinery [92]. Interestingly Ghanevati and Miller demonstrated that inhibition of the proteasome machinery in neuronal cultures leads to the progressive accumulation of phospho-*β*-catenin protein and formation of scattered, punctate cytoplasmic inclusions, which ultimately coalesced into a large cytoplasmic aggresome [92].

Striatal-enriched protein tyrosine phosphatase 61 (STEP61), the only isoform of this brain-specific family of phosphatases expressed in the cortex, localizes to postsynaptic terminals and the endoplasmic reticulum [93]. STEP61 associates with the NMDA receptor (NMDAR) complex, reduces NMDAR activity, and opposes the induction of LTP [93] with deleterious effects on cognitive functions. Kurup and colleagues demonstrated that STEP61 levels are elevated in aged transgenic AD model mice (Tg2576) and in AD brains and that A*β* is sufficient to increase STEP61 levels [93]. Increased STEP61 both in mice brain and in A*β*-treated cells had been found, thus suggesting an inhibition of the UPS [93]. The evidence outlined above highlights how a defective UPS activity is also associated with the accumulation of proteins, whose activation persists over the time, thus contributing to cognitive dysfunction in AD.

Studies have demonstrated that proteasome inhibition may occur also during normal aging [94, 95], which definitively represents one of the main risk factors for AD development. Thus, proteasome dysfunction together with other unknown mechanisms could contribute to protein aggregation throughout the lifetime. In that picture, the analysis of four molecular chaperones (Grp78, Grp94, PDI, and calnexin) revealed a marked decrease in aged rat hippocampus compared to young controls [96]. In addition, the levels of ubiquitinylated proteins were increased [96]. Thus, an age-related decrease in chaperone expression, together with an age-related decrease in proteasome activity [94, 97], conceivably could account for the increased content of ubiquitinylated proteins. Indeed, aged rats could be more predisposed to the formation of protein aggregates that in turn disrupt cellular functions and provide nucleation sites for the aggregation of other proteins. This scenario, together with environmental, genetic, and other unknown factors, could predispose development of age-related neurodegenerative disorders, such as AD.

### 2.5. Oxidative Modifications of UCHL1 in Alzheimer Disease

Protein ubiquitinylation can be regulated by modulating the enzyme levels or activity of deubiquitinylating enzymes such as UCHL1. This is an important mechanism for regulating a variety of cellular processes, including synaptic function, protein degradation, and neuronal apoptosis [98]. Thus, dysfunction of UCHL1 has been directly implicated in neurodegenerative diseases, such as AD. Of special interest is the finding that UCHL1 is involved, by maintenance of ubiquitinylation/deubiquitinylation machinery, in memory formation. In particular, the first study on UCHL1 was conducted in* Aplysia*, a model system used to investigate neuronal events associated with learning. Further studies confirmed the relevance of UCHL1 in synaptic function [99, 100].

Knockout mice for UCHL1 showed decreasing acetylcholine release from the synaptic terminal, which could be due to perturbed ubiquitin-dependent pathways as a result of decreased ubiquitin recycling. This reduction in content release is accompanied by hindered synaptic plasticity, nerve terminal retraction, and axonal degeneration [100]. In line with these findings, Zhang et al. showed that the gracile axonal dystrophy (gad) mutant mouse, which presents a deletion within the gene encoding for UCHL1, displays brain axonal degeneration [101]. These data suggest that the lower expression of UCHL1 may be partially responsible for cognitive impairment and Alzheimer pathophysiology. To consolidate these results and the involvement of UCHL1 in AD, Zhang and coworkers administered UCHL1 by intracranial injection of UCHL1-expressing rAAV into the hippocampus of the transgenic mice. Increased expression of the enzyme reduced A*β* production, inhibited neuritic plaque formation, and improved memory deficits [101]. A second study on a double transgenic mouse model of AD further supports this evidence. These mice showed cognitive defects such as inhibition of LTP, and the protein level of UCHL1 was significantly decreased in hippocampus [102]. Transduction to hippocampal slices of UCHL1 fused to the domain of HIV-transactivator protein (TAT) significantly restored A*β*-induced inhibition of LTP and also reestablishes normal UCH activity, basal neurotransmission, and synaptic plasticity and improves associative memory in APP/PS1 mice [102]. Interestingly, exogenous UCHL1 ameliorated *β*-amyloid-induced synaptic and memory dysfunction in an AD mouse model. UCHL1 is important at synapses and suggests that increased UCHL1 activity could counteract certain symptoms in AD.

Furthermore, data from the Butterfield group demonstrated that UCHL1 reduced activity was due to specific oxidative modifications, which can block its normal functioning. In fact an increased amount of oxidatively modified UCHL1 was found in the brains of AD patients, compared to normal brains [46, 103]. Proteomics analyses showed that UCHL1 is a major target of oxidative damage in frontal cortex of AD subjects [46], which is extensively modified by carbonyl formation, methionine oxidation, and cysteine oxidation [69]. Moreover, in the neurofibrillary tangles of AD patients a deposition of proteins modified by HNE, a product of lipid peroxidation, was reported [104]. To support this view,* in vitro *data showed that addition of HNE induced the HNE modification of recombinant UCHL1 [105]. UCHL1 immunostaining displayed a prominent association between the enzyme and neurofibrillary tangles and the level of soluble UCHL1 protein is inversely proportional to the number of tangles in AD brains [69]. Potentially due to its sequestration in neurofibrillary tangles, soluble UCHL1 levels are decreased in postmortem AD brains [37]. In inherited AD, UCHL1 was oxidatively damaged [106].

It is well known that oxidative stress causes protein modification, which can result in altered protein function. A reduction in the levels of functional UCHL1 was speculated to contribute to the pathogenesis of AD. In light of this evidence, the hydrolase activity of HNE-modified UCHL1 was reduced to about 40–80% of nonmodified UCHL1 and was inversely correlated with the degree of modification [46, 69]. A recent study from our laboratory observed that UCHL1 is a target of oxidative damage also in Down Syndrome (DS) brains [47]. DS presents many common features of AD, such as early deposition of A*β* plaques and, above 40 years of age, development of AD-like dementia [107]. Similar to what is found in AD, UCHL1 enzyme activity was decreased about 30% in DS brain compared to controls [47]. These two events, oxidation and decreased activity of UCHL1, can be correlated in DS subjects similar to AD. Moreover, in DS subjects UCHL1 impairment is an early event occurring before clinical manifestation of dementia, thus contributing to neurodegenerative phenomena.

Overall these results discussed in this review show that in different animal models and human specimens aberrant UCHL1 activity is caused by oxidative modifications that in turn might lead to dysfunction of the neuronal ubiquitinylation/deubiquitinylation machinery, causing synaptic deterioration and neuronal degeneration in AD (Figure 1). Moreover, overexpression of UCHL1 delays AD progression in mouse models, and* UCHL1* gene therapy, to overexpress UCHL1, in the brain potentially could be a promising disease-modifying strategy for AD therapeutics.

## 3. Conclusions 

A close connection between protein clearance network dysfunction and mechanisms of neurodegeneration is well documented. Potentially toxic oxidized and aggregated proteins harm neuronal cells once these are deprived of the cytoprotective functions of the PQC. In this scenario, a crucial role is played by oxidative stress that contributes to the buildup of oxidized/misfolded proteins. Concomitantly, oxidative stress targets members of the PQC, such as proteasome subunits and UCHL1, thus leading to its reduced ability to remove damaged/dysfunctional proteins. Taken together, these findings highlight that induction/protection of protein degradative system may represent an efficient therapeutic strategy for AD, as well as other neurodegenerative diseases.

## Abbreviations

AD: Alzheimer disease
APP: Amyloid-*β* protein precursor
A*β*: Amyloid-*β*- (A*β*-) peptide
ATP: Adenosine triphosphate
BACE1: *β*-secretase 1
DS: Down Syndrome
DUBs: Deubiquitinylating enzymes
E2: Ubiquitin-conjugating enzyme
E3: Ubiquitin ligase
ER: Endoplasmic reticulum
FBL2: F-box and leucine rich repeat protein 2
HECT: E6-AP carboxyl terminus
HNE: 4-Hydroxynonenal
HRD1: ERAD-associated E3 ubiquitin protein ligase
LTP: Long Term potentiation
Lys: Lysine
MCI: Mild cognitive impairment
NFT: Neurofibrillary tangles
NO: Nitric oxide
OTU: Ovarian tumor-like proteases
PDI: Protein disulphide isomerase
PGJ2: Prostaglandin J2
PHF: Paired helical filaments
PQC: Protein quality control system
PS1: Presenilin 1
ROS: Reactive oxygen species
SCF: Skp1–Cullin1–F-box protein
SP: Senile plaques
STEP61: Striatal-enriched protein tyrosine phosphatase 61
SUMO-1: Small ubiquitin-like modifier protein 1
UBB^+1^: Ubiquitin-B^+1^
UCH: Ubiquitin carboxyl-terminal hydrolases
UCHL1: Ubiquitin carboxyterminal hydrolase L1
UPS: Ubiquitin-proteasome system
UPR: Unfolded protein response
USP: Ubiquitin-specific proteases.

## References

[1] Cuanalo-Contreras K., Mukherjee A., Soto C. (2013). Role of protein misfolding and proteostasis deficiency in protein misfolding diseases and aging. *International Journal of Cell Biology*.

[2] Ortuño-Sahagún D., Pallàs M., Rojas-Mayorquín A. E. (2014). Oxidative stress in aging: advances in proteomic approaches. *Oxidative Medicine and Cellular Longevity*.

[3] Grune T., Reinheckel T., Davies K. J. A. (1997). Degradation of oxidized proteins in mammalian cells. *The FASEB Journal*.

[4] Dantuma N. P., Bott L. C. (2014). The ubiquitin-proteasome system in neurodegenerative diseases: precipitating factor, yet part of the solution. *Frontiers in Molecular Neuroscience*.

[5] Ciechanover A., Brundin P. (2003). The ubiquitin proteasome system in neurodegenerative diseases: sometimes the chicken, sometimes the egg. *Neuron*.

[6] Breusing N., Grune T. (2008). Regulation of proteasome-mediated protein degradation during oxidative stress and aging. *Biological Chemistry*.

[7] Höhn T. J. A., Grune T. (2014). The proteasome and the degradation of oxidized proteins: part III-Redox regulation of the proteasomal system. *Redox Biology*.

[8] Pandey U. B., Nie Z., Batlevi Y. (2007). HDAC6 rescues neurodegeneration and provides an essential link between autophagy and the UPS. *Nature*.

[9] Milani M., Rzymski T., Mellor H. R. (2009). The role of ATF4 stabilization and autophagy in resistance of breast cancer cells treated with Bortezomib. *Cancer Research*.

[10] Pan T., Kondo S., Zhu W., Xie W., Jankovic J., Le W. (2008). Neuroprotection of rapamycin in lactacystin-induced neurodegeneration via autophagy enhancement. *Neurobiology of Disease*.

[11] Korolchuk V. I., Menzies F. M., Rubinsztein D. C. (2010). Mechanisms of cross-talk between the ubiquitin-proteasome and autophagy-lysosome systems. *FEBS Letters*.

[12] Perluigi M., Di Domenico F., Butterfield D. A. (2015). mTOR signaling in aging and neurodegeneration: at the crossroad between metabolism dysfunction and impairment of autophagy. *Neurobiology of Disease*.

[13] Jung T., Grune T. (2008). The proteasome and its role in the degradation of oxidized proteins. *IUBMB Life*.

[14] Ciechanover A. (2015). The unravelling of the ubiquitin system. *Nature Reviews Molecular Cell Biology*.

[15] Sulistio Y. A., Heese K. (2015). The ubiquitin-proteasome system and molecular chaperone deregulation in Alzheimer's disease. *Molecular Neurobiology*.

[16] Huang Q., Figueiredo-Pereira M. E. (2010). Ubiquitin/proteasome pathway impairment in neurodegeneration: therapeutic implications. *Apoptosis*.

[17] Lim K.-L., Tan J. M. M. (2007). Role of the ubiquitin proteasome system in Parkinson's disease. *BMC Biochemistry*.

[18] Grimm S., Höhn A., Grune T. (2012). Oxidative protein damage and the proteasome. *Amino Acids*.

[19] Jung T., Catalgol B., Grune T. (2009). The proteasomal system. *Molecular Aspects of Medicine*.

[20] Blalock E. M., Geddes J. W., Chen K. C., Porter N. M., Markesbery W. R., Landfield P. W. (2004). Incipient Alzheimer's disease: microarray correlation analyses reveal major transcriptional and tumor suppressor responses. *Proceedings of the National Academy of Sciences of the United States of America*.

[21] Watanabe T., Hikichi Y., Willuweit A., Shintani Y., Horiguchi T. (2012). FBL2 regulates amyloid precursor protein (APP) metabolism by promoting ubiquitination-dependent APP degradation and inhibition of APP endocytosis. *Journal of Neuroscience*.

[22] Koegl M., Hoppe T., Schlenker S., Ulrich H. D., Mayer T. U., Jentsch S. (1999). A novel ubiquitination factor, E4, is involved in multiubiquitin chain assembly. *Cell*.

[23] Thrower J. S., Hoffman L., Rechsteiner M., Pickart C. M. (2000). Recognition of the polyubiquitin proteolytic signal. *The EMBO Journal*.

[24] Bennett E. J., Harper J. W. (2008). DNA damage: ubiquitin marks the spot. *Nature Structural and Molecular Biology*.

[25] Hislop J. N., von Zastrow M. (2011). Role of ubiquitination in endocytic trafficking of G-protein-coupled receptors. *Traffic*.

[26] Raiborg C., Stenmark H. (2009). The ESCRT machinery in endosomal sorting of ubiquitylated membrane proteins. *Nature*.

[27] Kim J. H., Park K. C., Chung S. S., Bang O., Chung C. H. (2003). Deubiquitinating enzymes as cellular regulators. *Journal of Biochemistry*.

[28] Di Domenico F., Head E., Butterfield D. A., Perluigi M. (2014). Oxidative stress and proteostasis network: culprit and casualty of Alzheimer's-like neurodegeneration. *Advances in Geriatrics*.

[29] Berlett B. S., Stadtman E. R. (1997). Protein oxidation in aging, disease, and oxidative stress. *The Journal of Biological Chemistry*.

[30] Butterfield D. A., Stadtman E. R. (1997). Protein oxidation processes in aging brain. *Advances in Cell Aging and Gerontology*.

[31] Davies K. J. (1987). Protein damage and degradation by oxygen radicals. I. general aspects. *The Journal of Biological Chemistry*.

[32] Cervera J., Levine R. L. (1988). Modulation of the hydrophobicity of glutamine synthetase by mixed-function oxidation. *The FASEB Journal*.

[33] Davies K. J. A. (2001). Degradation of oxidized proteins by the 20S proteasome. *Biochimie*.

[34] Wilkinson K. D. (1997). Regulation of ubiquitin-dependent processes by deubiquitinating enzymes. *The FASEB Journal*.

[35] Fang Y., Fu D., Shen X.-Z. (2010). The potential role of ubiquitin c-terminal hydrolases in oncogenesis. *Biochimica et Biophysica Acta*.

[36] Jara J. H., Frank D. D., Özdinler P. H. (2013). Could dysregulation of UPS be a common underlying mechanism for cancer and neurodegeneration? Lessons from UCHL1. *Cell Biochemistry and Biophysics*.

[37] Setsuie R., Wada K. (2007). The functions of UCH-L1 and its relation to neurodegenerative diseases. *Neurochemistry International*.

[38] Das C., Hoang Q. Q., Kreinbring C. A. (2006). Structural basis for conformational plasticity of the Parkinson's disease-associated ubiquitin hydrolase UCH-L1. *Proceedings of the National Academy of Sciences of the United States of America*.

[39] Larsen C. N., Krantz B. A., Wilkinson K. D. (1998). Substrate specificity of deubiquitinating enzymes: ubiquitin C-terminal hydrolases. *Biochemistry*.

[40] Liu Y., Fallon L., Lashuel H. A., Liu Z., Lansbury P. T. (2002). The UCH-L1 gene encodes two opposing enzymatic activities that affect *α*-synuclein degradation and Parkinson's disease susceptibility. *Cell*.

[41] Osaka H., Wang Y.-L., Takada K. (2003). Ubiquitin carboxy-terminal hydrolase L1 binds to and stabilizes monoubiquitin in neuron. *Human Molecular Genetics*.

[42] Proctor C. J., Tangeman P. J., Ardley H. C. (2010). Modelling the role of UCH-L1 on protein aggregation in age-related neurodegeneration. *PLoS ONE*.

[43] Shen H., Sikorska M., Leblanc J., Walker P. R., Liu Q. Y. (2006). Oxidative stress regulated expression of ubiquitin Carboxyl-terminal Hydrolase-L1: role in cell survival. *Apoptosis*.

[44] Nishikawa K., Li H., Kawamura R. (2003). Alterations of structure and hydrolase activity of parkinsonism-associated human ubiquitin carboxyl-terminal hydrolase L1 variants. *Biochemical and Biophysical Research Communications*.

[45] Castegna A., Thongboonkerd V., Klein J. (2004). Proteomic analysis of brain proteins in the gracile axonal dystrophy (gad) mouse, a syndrome that emanates from dysfunctional ubiquitin carboxyl-terminal hydrolase L-1, reveals oxidation of key proteins. *Journal of Neurochemistry*.

[46] Castegna A., Aksenov M., Aksenova M. (2002). Proteomic identification of oxidatively modified proteins in Alzheimer's disease brain. Part I: creatine kinase BB, glutamine synthase, and ubiquitin carboxy-terminal hydrolase L-1. *Free Radical Biology and Medicine*.

[47] Di Domenico F., Coccia R., Cocciolo A. (2013). Impairment of proteostasis network in Down syndrome prior to the development of Alzheimer's disease neuropathology: redox proteomics analysis of human brain. *Biochimica et Biophysica Acta*.

[48] Kabuta T., Furuta A., Aoki S., Furuta K., Wada K. (2008). Aberrant interaction between Parkinson disease-associated mutant UCH-L1 and the lysosomal receptor for chaperone-mediated autophagy. *The Journal of Biological Chemistry*.

[49] Kabuta T., Setsuie R., Mitsui T. (2008). Aberrant molecular properties shared by familial Parkinson's disease-associated mutant UCH-L1 and carbonyl-modified UCH-L1. *Human Molecular Genetics*.

[50] Cole G. M., Timiras P. S. (1987). Ubiquitin-protein conjugates in Alzheimer's lesions. *Neuroscience Letters*.

[51] Perry G., Friedman R., Shaw G., Chau V. (1987). Ubiquitin is detected in neurofibrillary tangles and senile plaque neurites of Alzheimer disease brains. *Proceedings of the National Academy of Sciences of the United States of America*.

[52] Mori H., Kondo J., Ihara Y. (1987). Ubiquitin is a component of paired helical filaments in Alzheimer's disease. *Science*.

[53] Gregori L., Fuchs C., Figueiredo-Pereira M. E., Van Nostrand W. E., Goldgaber D. (1995). Amyloid *β*-protein inhibits ubiquitin-dependent protein degradation in vitro. *The Journal of Biological Chemistry*.

[54] Keller J. N., Hanni K. B., Markesbery W. R. (2000). Impaired proteasome function in Alzheimer's disease. *Journal of Neurochemistry*.

[55] Oh S., Hong H. S., Hwang E. (2005). Amyloid peptide attenuates the proteasome activity in neuronal cells. *Mechanisms of Ageing and Development*.

[56] Cecarini V., Bonfili L., Amici M., Angeletti M., Keller J. N., Eleuteri A. M. (2008). Amyloid peptides in different assembly states and related effects on isolated and cellular proteasomes. *Brain Research*.

[57] Rosen K. M., Moussa C. E.-H., Lee H.-K. (2010). Parkin reverses intracellular *β*-amyloid accumulation and its negative effects on proteasome function. *Journal of Neuroscience Research*.

[58] Hong X., Liu J., Zhu G. (2014). Parkin overexpression ameliorates hippocampal long-term potentiation and beta-amyloid load in an Alzheimer's disease mouse model. *Human Molecular Genetics*.

[59] Kaneko M., Koike H., Saito R., Kitamura Y., Okuma Y., Nomura Y. (2010). Loss of HRD1-mediated protein degradation causes amyloid precursor protein accumulation and amyloid-*β* generation. *The Journal of Neuroscience*.

[60] Zhang M., Deng Y., Luo Y. (2012). Control of BACE1 degradaton and APP processing by ubiquitin carboxyl-terminal hydrolase L1. *Journal of Neurochemistry*.

[61] Butterfield D. A., Perluigi M., Sultana R. (2006). Oxidative stress in Alzheimer's disease brain: new insights from redox proteomics. *European Journal of Pharmacology*.

[62] Petropoulos I., Conconi M., Wang X. (2000). Increase of oxidatively modified protein is associated with a decrease of proteasome activity and content in aging epidermal cells. *Journals of Gerontology—Series A: Biological Sciences and Medical Sciences*.

[63] Starke-Reed P. E., Oliver C. N. (1989). Protein oxidation and proteolysis during aging and oxidative stress. *Archives of Biochemistry and Biophysics*.

[64] Szweda P. A., Friguet B., Szweda L. I. (2002). Proteolysis, free radicals, and aging. *Free Radical Biology and Medicine*.

[65] Reinheckel T., Sitte N., Ullrich O., Kuckelkorn U., Davies K. J. A., Grune T. (1998). Comparative resistance of the 20S and 26S proteasome to oxidative stress. *Biochemical Journal*.

[66] Rivett A. J. (1986). Regulation of intracellular protein turnover: covalent modification as a mechanism of marking proteins for degradation. *Current Topics in Cellular Regulation*.

[67] Sitte N., Huber M., Grune T. (2000). Proteasome inhibition by lipofuscin/ceroid during postmitotic aging of fibroblasts. *The FASEB Journal*.

[68] Sultana R., Boyd-Kimball D., Poon H. F. (2006). Redox proteomics identification of oxidized proteins in Alzheimer's disease hippocampus and cerebellum: an approach to understand pathological and biochemical alterations in AD. *Neurobiology of Aging*.

[69] Choi J., Levey A. I., Weintraub S. T. (2004). Oxidative modifications and down-regulation of ubiquitin carboxyl-terminal hydrolase L1 associated with idiopathic Parkinson's and Alzheimer's diseases. *The Journal of Biological Chemistry*.

[70] Saito R., Kaneko M., Kitamura Y. (2014). Effects of oxidative stress on the solubility of HRD1, a ubiquitin ligase implicated in Alzheimer's disease. *PLoS ONE*.

[71] Castegna A., Aksenov M., Thongboonkerd V. (2002). Proteomic identification of oxidatively modified proteins in Alzheimer's disease brain. Part II: dihydropyrimidinase-related protein 2, alpha-enolase and heat shock cognate 71. *Journal of Neurochemistry*.

[72] Uehara T., Nakamura T., Yao D. (2006). S-nitrosylated protein-disulphide isomerase links protein misfolding to neurodegeneration. *Nature*.

[73] Nakamura T., Prikhodko O. A., Pirie E. (2015). Aberrant protein *S*-nitrosylation contributes to the pathophysiology of neurodegenerative diseases. *Neurobiology of Disease*.

[74] Cecarini V., Ding Q., Keller J. N. (2007). Oxidative inactivation of the proteasome in Alzheimer's disease. *Free Radical Research*.

[75] van Leeuwen F. W., de Kleijn D. P. V., van den Hurk H. H. (1998). Frameshift mutants of *β* amyloid precursor protein and ubiquitin-B in Alzheimer's and Down patients. *Science*.

[76] Lam Y. A., Pickart C. M., Alban A. (2000). Inhibition of the ubiquitin-proteasome system in Alzheimer's disease. *Proceedings of the National Academy of Sciences of the United States of America*.

[77] De Vrij F. M. S., Sluijs J. A., Gregori L. (2001). Mutant ubiquitin expressed in Alzheimer's disease causes neuronal death. *The FASEB Journal*.

[78] Hope A. D., De Silva R., Fischer D. F., Hol E. M., Van Leeuwen F. W., Lees A. J. (2003). Alzheimer's associated variant ubiquitin causes inhibition of the 26S proteasome and chaperone expression. *Journal of Neurochemistry*.

[79] Song S., Kim S.-Y., Hong Y.-M. (2003). Essential role of E2-25K/Hip-2 in mediating amyloid-*β* neurotoxicity. *Molecular Cell*.

[80] Chen Z., Pickart C. M. (1990). A 25-kilodalton ubiquitin carrier protein (E2) catalyzes multi-ubiquitin chain synthesis via lysine 48 of ubiquitin. *The Journal of Biological Chemistry*.

[81] van Tijn P., Dennissen F. J. A., Gentier R. J. G. (2012). Mutant ubiquitin decreases amyloid beta plaque formation in a transgenic mouse model of Alzheimer's disease. *Neurochemistry International*.

[82] Tai H.-C., Serrano-Pozo A., Hashimoto T., Frosch M. P., Spires-Jones T. L., Hyman B. T. (2012). The synaptic accumulation of hyperphosphorylated tau oligomers in alzheimer disease is associated with dysfunction of the ubiquitin-proteasome system. *American Journal of Pathology*.

[83] Zhang J. Y., Liu S. J., Li H. L., Wang J.-Z. (2005). Microtubule-associated protein tau is a substrate of ATP/Mg^2+^-dependent proteasome protease system. *Journal of Neural Transmission*.

[84] Cripps D., Thomas S. N., Jeng Y., Yang F., Davies P., Yang A. J. (2006). Alzheimer disease-specific conformation of hyperphosphorylated paired helical filament-Tau is polyubiquitinated through Lys-48, Lys-11, and Lys-6 ubiquitin conjugation. *The Journal of Biological Chemistry*.

[85] Gillardon F., Kloß A., Berg M. (2007). The 20S proteasome isolated from Alzheimer's disease brain shows post-translational modifications but unchanged proteolytic activity. *Journal of Neurochemistry*.

[86] Shen Y. X., Sun A. M., Fang S. (2012). Hrd1 facilitates tau degradation and promotes neuron survival. *Current Molecular Medicine*.

[87] Poppek D., Keck S., Ermak G. (2006). Phosphorylation inhibits turnover of the tau protein by the proteasome: influence of RCAN1 and oxidative stress. *Biochemical Journal*.

[88] Shimura H., Schwartz D., Gygi S. P., Kosik K. S. (2004). CHIP-Hsc70 complex ubiquitinates phosphorylated tau and enhances cell survival. *The Journal of Biological Chemistry*.

[89] Petrucelli L., Dickson D., Kehoe K. (2004). CHIP and Hsp70 regulate tau ubiquitination, degradation and aggregation. *Human Molecular Genetics*.

[90] Grune T., Botzen D., Engels M. (2010). Tau protein degradation is catalyzed by the ATP/ubiquitin-independent 20S proteasome under normal cell conditions. *Archives of Biochemistry and Biophysics*.

[91] Huang Q., Wang H., Perry S. W., Figueiredo-Pereira M. E. (2013). Negative regulation of 26S proteasome stability via calpain-mediated cleavage of Rpn10 subunit upon mitochondrial dysfunction in neurons. *The Journal of Biological Chemistry*.

[92] Ghanevati M., Miller C. A. (2005). Phospho-beta-catenin accumulation in Alzheimer's disease and in aggresomes attributable to proteasome dysfunction. *Journal of Molecular Neuroscience*.

[93] Kurup P., Zhang Y., Xu J. (2010). A*β*-mediated NMDA receptor endocytosis in alzheimer's disease involves ubiquitination of the tyrosine phosphatase STEP61. *The Journal of Neuroscience*.

[94] Keller J. N., Hanni K. B., Markesbery W. R. (2000). Possible involvement of proteasome inhibition in aging: implications for oxidative stress. *Mechanisms of Ageing and Development*.

[95] Keller J. N., Gee J., Ding Q. (2002). The proteasome in brain aging. *Ageing Research Reviews*.

[96] Paz Gavilán M., Vela J., Castaño A. (2006). Cellular environment facilitates protein accumulation in aged rat hippocampus. *Neurobiology of Aging*.

[97] Keller J. N., Huang F. F., Markesbery W. R. (2000). Decreased levels of proteasome activity and proteasome expression in aging spinal cord. *Neuroscience*.

[98] Goder V. (2012). Roles of ubiquitin in endoplasmic reticulum-associated protein degradation (ERAD). *Current Protein and Peptide Science*.

[99] Cartier A. E., Djakovic S. N., Salehi A., Wilson S. M., Masliah E., Patrick G. N. (2009). Regulation of synaptic structure by ubiquitin C-terminal hydrolase L1. *The Journal of Neuroscience*.

[100] Chen F., Sugiura Y., Myers K. G., Liu Y., Lin W. (2010). Ubiquitin carboxyl-terminal hydrolase L1 is required for maintaining the structure and function of the neuromuscular junction. *Proceedings of the National Academy of Sciences of the United States of America*.

[101] Zhang M., Cai F., Zhang S., Zhang S., Song W. (2014). Overexpression of ubiquitin carboxyl-terminal hydrolase L1 (UCHL1) delays Alzheimer's progression in vivo. *Scientific Reports*.

[102] Gong B., Cao Z., Zheng P. (2006). Ubiquitin hydrolase Uch-L1 rescues beta-amyloid-induced decreases in synaptic function and contextual memory. *Cell*.

[103] Butterfield D. A. (2006). Oxidative stress in neurodegenerative disorders. *Antioxidants and Redox Signaling*.

[104] Montine K. S., Kim P. J., Olson S. J., Markesbery W. R., Montine T. J. (1997). 4-Hydroxy-2-nonenal pyrrole adducts in human neurodegenerative disease. *Journal of Neuropathology and Experimental Neurology*.

[105] Castellani R. J., Perry G., Siedlak S. L. (2002). Hydroxynonenal adducts indicate a role for lipid peroxidation in neocortical and brainstem Lewy bodies in humans. *Neuroscience Letters*.

[106] Butterfield D. A., Poon H. F., St. Clair D. (2006). Redox proteomics identification of oxidatively modified hippocampal proteins in mild cognitive impairment: insights into the development of Alzheimer's disease. *Neurobiology of Disease*.

[107] Perluigi M., Di Domenico F., Buttterfield D. A. (2014). Unraveling the complexity of neurodegeneration in brains of subjects with Down syndrome: insights from proteomics. *Proteomics—Clinical Applications*.

